# Disclosure of HIV status to sexual partner and its associated factors among pregnant women living with HIV attending prenatal care in Amhara Regional state Referral Hospitals, Ethiopia

**DOI:** 10.1371/journal.pone.0280045

**Published:** 2023-01-17

**Authors:** Nebiyu Solomon Tibebu, Bayew Kelkay Rade, Azmeraw Ambachew Kebede, Belayneh Ayanaw Kassie

**Affiliations:** 1 Department of Clinical Midwifery, School of Midwifery, College of Medicine and Health Sciences, University of Gondar, Gondar, Ethiopia; 2 Department of General Midwifery, School of Midwifery, College of Medicine and Health Sciences, University of Gondar, Gondar, Ethiopia; 3 Department of Women’s and Family Health, School of Midwifery, College of Medicine and Health Sciences, University of Gondar, Gondar, Ethiopia; University of California San Francisco, UNITED STATES

## Abstract

**Background:**

Disclosure of Human Immunodeficiency Virus (HIV) status to sexual partners plays a significant role in the successful prevention and care of HIV infection. Pregnant women who did not reveal their HIV status to their sexual partners make the prevention and control efforts challenging. Therefore, this study was aimed to assess HIV status disclosure to sexual partners and associated factors among pregnant women living with HIV attending prenatal care in Amhara Regional state referral Hospitals, Ethiopia in 2021.

**Methods:**

An institution-based cross-sectional study was conducted from October 17^th^, 2020 to March 1^st^, 2021. A total of 423 pregnant women living with HIV were participated in this study. A systematic random sampling technique was used to select all eligible women. Data was collected using a semi-structured, pretested, and interviewer-administered questionnaire. EPI INFO version 7 and SPSS version 21 were used for data entry and analysis, respectively. Both univariable and multivariable logistic regression analyses were performed to find factors associated with women’s disclosure status to a sexual partner. Statistical association was decided based on the adjusted odds ratio (AOR) with its 95% Confidence Interval (CI) and p-value of ≤ 0.05.

**Results:**

The prevalence of disclosure of their HIV status to their sexual partners was 73% (95% CI: 68.9%, 77.3%). Being an urban resident (AOR = 5.04, 95% CI: 2.14, 11.81), diagnosed HIV before pregnancy (AOR = 7.77, 95% CI: 3.09, 19.52), disclosing their HIV status to others (AOR = 7.01, 95% CI: 3.78, 13.25), planned pregnancy (AOR = 2.46, 95% CI: 1.32, 4.57), and having good knowledge on HIV/AIDS prevention (AOR = 2.19, 95% CI:1.22, 3.94) were found to be statistically significant with women’s disclosure of their HIV status to their sexual partner.

**Conclusion:**

In this study, nearly three-fourth of pregnant women disclosed their HIV status to their sexual partner. Thus, setting strategies in preventing unplanned pregnancy, HIV diagnosed before pregnancy, and increasing knowledge of HIV prevention will have significant role in escalating women’s disclosure status.

## Introduction

Human Immunodeficiency Virus/Acquired immune deficiency syndrome (HIV/AIDS) is a major public health problem globally. Nearly 37.6 million people across the globe live with HIV/AIDS [1]. Women carry an unbalanced global burden of HIV infection in which 25.7 million people are living with HIV in Africa; of these, 12 million were women [1, 2]. In 2018, 63.08% of 650,000 infected adults were women in Ethiopia [3]. On the other hand, a systematic review and meta-analysis revealed that the prevalence of HIV among pregnant women was 5.74% [4].

The prevention and control of HIV infection depends on the successful prevention of new infections and by treating currently infected individuals [5]. Disclosure of HIV status and prevention of mother-to-child transmission (PMTCT) are both important for the prevention and control of HIV increments [6]. Disclosure is the process of revealing HIV positive status to sexual partner (s), family members, friends or others in their social circle, but not to health care providers [7]. Besides, disclosure of HIV status is a unique strategy that fulfills these dual goals [8, 9].

The level of HIV positive status disclosure in the developed world (average 71%; range: 42%-100%) was higher compared to the developing world report (average 52%; range: 16.7%-86%) [5]. The frequency of HIV disclosure in Sub-Saharan Africa (SSA) among women ranges from 16.7% to 86% and around 63.9% of pregnant women disclosed their HIV status to sexual partners [6, 10]. Studies in Ethiopia revealed that the pooled prevalence of HIV status disclosure to sexual partners among individuals receiving HIV care was 73% [11], and the prevalence of pregnant women’s disclosure of their HIV status to their partners were 51.7% and 73% two studies in Addis Ababa [12, 13], and 89.7% Northwest Ethiopia [14].

Disclosure of HIV status to a sexual partner is an important prevention strategy as emphasized by the World Health Organization (WHO) and centers for disease control (CDC) to effectively prevent MTCT [15, 16]. Disclosure of HIV-positive status to sexual partners, close relatives and other important persons provides several benefits to the infected person and the community at large [16]. Furthermore, it may motivate sexual partners to seek testing, change their behavior, decrease the transmission and improve the management of HIV [5, 17]. Women who disclose their status to their partners are more likely to take part in PMTCT programs [18]. Besides, they can communicate freely for their reproductive health and limit the number of unintended pregnancies, cessation of breastfeeding, help them to take the recommended antiretroviral (ARV) drugs to herself and her newborn appropriately, able to access available support services, help to create a sense of closeness in the family, reduce feelings of anxiety and relieve the burden of living with the secret of being HIV-positive [5, 6]. Despite these many benefits, women who disclose their status include loss of economic support, abandonment, blame, physical and emotional abuse, discrimination and disruption of family relations [5, 19]. Thus, couple screening, progressive counseling and overcoming the problem of stigma are the approaches for enhancing disclosure and prevention of HIV transmission [20, 21].

Non-disclosure of HIV-positive status to male partners has been identified as a key obstacle to HIV care engagement. It contributes to the risk of infant HIV acquisition and may deter use of PMTCT interventions and the greater risk of anxiety and depression [22, 23]. Male partners play a pivotal role in decision-making within the household including access to and use of healthcare services [24]. Hence, male partner involvement handles the prevention of infant HIV acquisition by deciding uptake of PMTCT interventions such as hospital visits for PMTCT, acceptance of ART (antiretroviral therapy), infant prophylaxis, and adherence to safe feeding options for the infant [22, 24, 25].

Despite the government of Ethiopia had executed lots of interventions to aware the community, reduce stigma, discrimination, social support, HIV testing, and counseling program to ease disclosure of HIV status, still the issue continued to be a major public health problem. Therefore, the purpose of this study was to assess the prevalence and there is paucity of evidence on factors associated with HIV status disclosure to sexual partners among pregnant women living with HIV attending ANC in Amhara Region Referral hospitals.

## Methods

### Study design, area, and period

An institution-based cross-sectional study was conducted in Referral Hospitals found in Amhara regional state, from October 17^th^, 2020 to March 1^st^, 2021. There are six referral Hospitals, namely the University of Gondar (UoG) Comprehensive Specialized Hospital, Felege Hiwot, Tibebe Giyon, Debremarkos, Dessie, and Debrebirhan Referral Hospitals, and two general Hospitals upgraded recently to referral Hospitals. Amhara region is the second-largest and most populous regional state in Ethiopia. It has a total of 19.2 million people. It is one of the hardest HIV/AIDS hit regions in the country. The prevalence of HIV among adults and pregnant women in this region is 1.5% and 0.8% respectively [26].

### Study population

All pregnant women living with HIV attended ANC follow-up in Amhara Region Referral Hospitals during the study period.

### Sample size determination and sampling procedure

**Fig 1:**
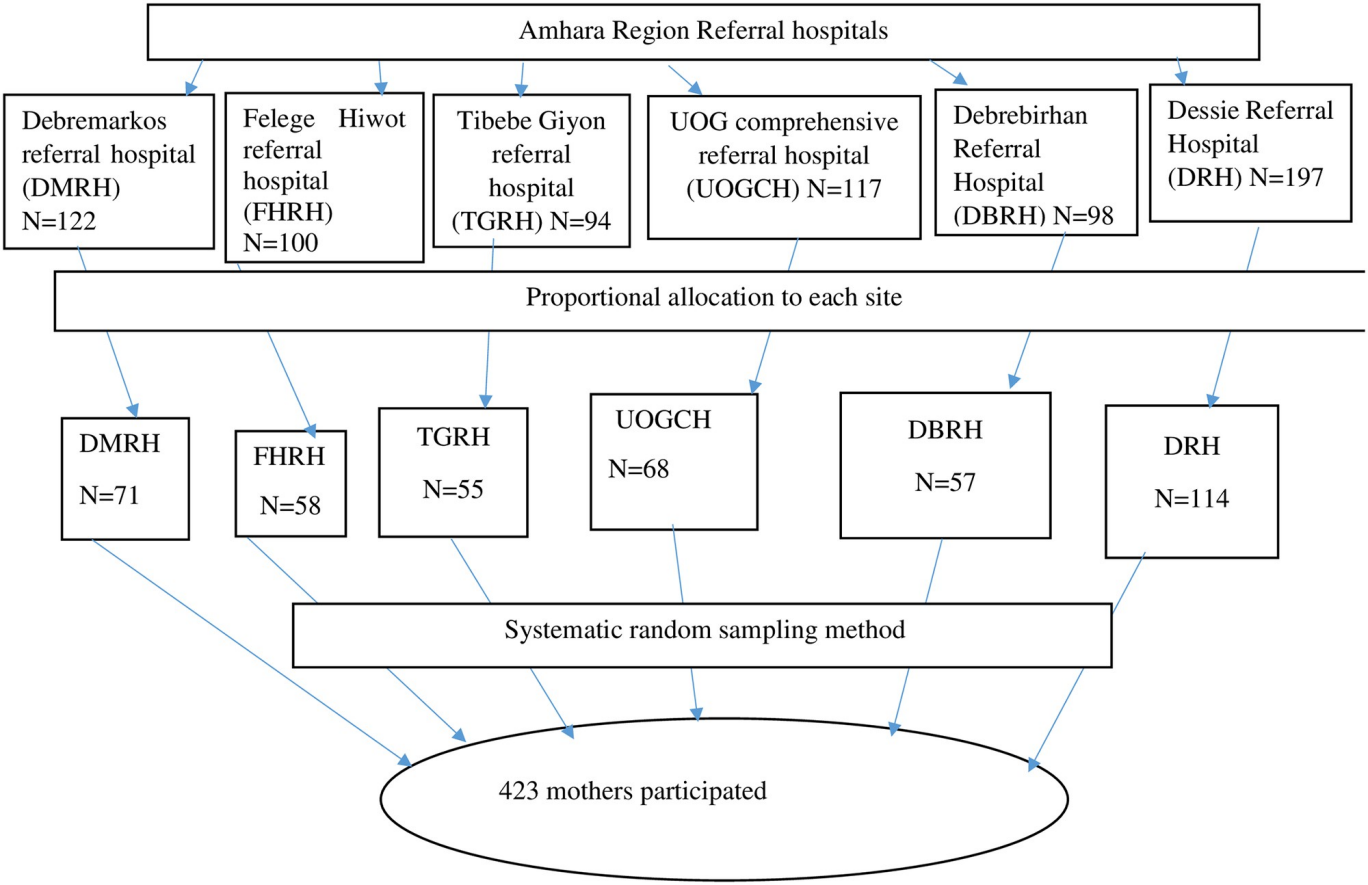
Schematic presentation of the sampling procedure done on disclosure status to sexual partners among HIV-positive pregnant women attending ANC service in Amhara Region Referral Hospitals, Ethiopia, 2020/2021.

The sample size for this study was determined using a single population proportion formula ($n=\frac{{\left(Z\alpha /2\right)}^{2}p\left(1-p\right)}{d^{2}}$) by considering the following assumptions; prevalence of HIV-positive pregnant women disclosure status to sexual partner was 51.7% [12], 95% level of confidence, and 5% margin of error. Therefore, where n = required sample size, α = level of significant, z = standard normal distribution curve value for 95% confidence level = 1.96, p = proportion of disclosure status among HIV-positive women to sexual partners, and d = margin of error. Finally, by considering a 10% non-response rate, the sample size was 423. The study was done in six referral Hospitals in Amhara Regional State. Using the PMTCT registration book as sampling frame, the sample size was distributed to the respective hospitals proportionally. Then, the samples were selected using systematic random sampling methods [Fig 1].

### Study variables

HIV Disclosure status to a sexual partner was the dependent variable whereas the age of the women, residence, educational status of women, family income, women’s occupation, religion, marital status, discussing for HIV with a sexual partner, partner’s HIV status, seen HIV positive person, seen mistreatment of PLWHA, educational status of the sexual partner, occupational status of sexual partner, number of pregnancies, number of alive children, planned pregnancy, ANC visits, time of tested positive for HIV status, duration of knowing their HIV status, currently on ART treatment and duration, place of HIV test, getting counseling before the test, getting counseling after test, getting ongoing counseling every visit, being a member of an association of PLWHA, presence of any infected family, disclosing HIV status to others, knowledge on HIV transmission and prevention, and attitude towards HIV.

### Operational definitions

**Disclosure of HIV status to sexual partner**: is revealing HIV-positive status of an infected person to a sexual partner [7].
**Sexual partner**: defined as the women’s current male sexual partner who is the father of her current pregnancy [27].
**Good knowledge**: women who correctly responded to three or more knowledge questions out of a total five items [28].
**Favorable attitude**: women who had a score of 16 to 25 attitude related questions were considered as high attitude towards HIV/AIDS [29].

### Data collection tools and procedure

The data collection tool was developed by reviewing related literature [12, 14, 15, 22, 29–31]. A semi-structured questionnaire was employed to collect the data through face-to-face interviews. Socio-demographic characteristics, obstetrics, and clinical characteristics, knowledge, and attitude towards HIV prevention and transmission related questions, were incorporated in the study tool. Six midwives collected the data and three master’s holders supervised the data collection. Content and face validation were done to improve the quality of assessment tool.

### Data quality controls

The questionnaire was first prepared in English and translated to the local language (Amharic) and back to English to keep its consistency and re-adjustments of inconsistent and inaccurate data were done accordingly. Before the actual data collection, a pretest was done on 5% of the sample size to check the response, language clarity, understanding of data collectors, and supervisors. The training was given for half a day for a better understanding of the overall process of the data collection procedure before data collection. During the actual data collection period, the questionnaire was checked for completeness daily by the supervisors and the principal investigator.

### Data processing and analysis

Data cleaning and cross-checking were done before data analysis. Data were checked, coded, and entered to EPI INFO version 7; then it was exported to Statistical Package for the Social Science (SPSS) version 21 for analysis (IBM^®^ SPSS^®^ Statistics 21). Both descriptive (frequency with percent, central tendency and dispersion) and analytic statistical procedures were done. Univariable binary logistic regression was used to identify statistically significant independent variables and variables having a p-value less than 0.2 used for the multivariable logistic regression analysis for controlling of possible confounders. In the multivariable binary logistic regression analysis, a p-value of <0.05 with a 95% confidence interval for the adjusted odds ratio was used to find the significance association. Model fitness for the final model was checked using Hosmer and Lemeshow goodness of fit.

### Ethical considerations

The ethical approval letter was obtained from the School of Midwifery under the delegation of the University of Gondar Institution Review Board (IRB) (R.No: SMIDW/18/2013 E.C) and a formal letter of study approval was obtained from each referral hospital. Then, the study participants were informed about the purpose of the study, the importance of their participation, and their rights. And then, written informed consent was obtained before the actual data collection.

## Result

### Socio-demographic related characteristics

A total of 423 HIV-positive pregnant women were interviewed, with a response rate of 100%. The mean age of the study participants was 29.61 years old (SD ±5.01) and 63.3% of the participants’ age was between the age group of 25-34-year-old. Two-thirds (65%) of the respondents were Orthodox Christian by religion and 80.1% of them lived in the urban areas. About 129 (30.5%) of them had completed secondary education. Of the total study participants, 301(71.2%) were married. One-quarter of (24.3%) the study participants were housewives by occupation [Table 1].

**Table 1:** Socio-demographic characteristics of study participants in Amhara Region Referral Hospitals, Ethiopia.

Characteristics	Frequency (N = 423)	Percent
**Age of women in years**		
18–24	79	18.7
25–34	268	63.3
35 and above	76	18.0
**Residence**		
Rural	84	19.9
Urban	339	80.1
**Religion**		
Orthodox Christian	275	65.0
Muslim	124	29.3
Protestant	24	5.7
**Women’s educational status**		
Can’t read and write	56	13.2
Can read and write	73	17.3
Primary (1–8)	75	17.7
Secondary (9–12)	129	30.5
College and above	90	21.3
**Women’s occupation**		
Housewife	101	24.8
Merchant	65	15.3
Government employee	64	15.0
Employee private organization	55	13.0
NGO employee	47	11.0
Farmer	61	14.4
Others ^a^	30	7.0
**Marital status**		
Married	301	71.2
Separated	56	13.2
Single	40	9.5
Divorced	26	6.1
**Sexual partner’s occupation (N = 357)**		
Merchant	122	34.1
NGO employee	30	8.4
Government employee	72	20.2
Daily laborer	46	12.9
Farmer	43	12.0
Private organization	44	12.4
**Average monthly family income**		
≤600 ETB	146	34.5
601–1601 ETB	74	17.5
1602–2602 ETB	91	21.5
≥2603 ETB	112	26.5

^a^ = students and daily laborer

NGO; Non-governmental Organization, ETB; Ethiopian Birr

### Obstetrics related characteristics

Of the total study participants, 290 (65.6%) were multigravida. Half of the study participants (52.7%) come for their second ANC visits. Regarding pregnancy planning, 329(77.8%) had planned pregnancy [Table 2].

**Table 2:** Obstetrics characteristics of study participants in Amhara Region Referral Hospitals, Ethiopia.

Characteristics	Frequency (N = 423)	Percentage
**Pregnancy number**		
prim gravida	133	31.4
Multigravida (≥ 2 pregnancy)	290	68.6
**Number of alive children**		
0	144	34.0
1	136	32.2
≥2	143	33.8
**Planned pregnancy**		
Yes	329	77.8
No	94	22.2
**ANC visit**		
First	23	5.4
Second	200	52.7
Third	158	37.4
Fourth and above	42	9.9

ANC; Antenatal Care

### Clinical related characteristics

From the total study participants, 311 (73.5%) knew their HIV status during pregnancy. Besides, around 282 (66.7%) of the study participants knew their HIV-positive status in more than six months. Three hundred twenty-six (77.1%) women were tested for HIV at the government health facility. Many of the study participants (94.3%) had counseling after getting their HIV test. About (60.8%) of the study participants were alone when they were tested. Most of 407(96.2%) were currently on ART, and 227(53.7%) participants were on ART for more than one year. The majority 378 (89.4%) seen HIV-positive persons, and one quarter of (25.8%) of the study participants have seen the mistreatment of PLWHA. About 370 (87.5) had a favorable attitude. Whereas around half of study participants 222 (52.5%) had good knowledge on HIV transmission and prevention towards HIV/AIDS [Table 3].

**Table 3:** Clinical characteristics of study participants and women’s knowledge on transmission, prevention, and attitude towards HIV in Amhara Region Referral Hospitals, Ethiopia.

Characteristics	Frequency (N = 423)	Percent
**Time of tested positive for HIV status**		
During pregnancy	311	73.5
Before pregnancy	112	26.5
**With whom when you test HIV**		
Alone	233	55.1
With their current sexual partner	147	34.8
Others ^b^	43	10.2
**Duration of known positive for HIV**		
Less than 6 months	141	33.3
6 months and more	282	66.7
**Place of HIV screening**		
Government Health facility	326	77.1
Private clinic	66	15.6
Freestanding VCT centres	31	7.3
**Counselled before test**		
Yes	225	53.2
No	198	46.8
**Counselled after test**		
Yes	399	94.3
No	24	5.7
**Got ongoing counselling every visit at the health facilities**		
Yes	353	83.5
No	70	16.5
**Currently on ART**		
Yes	407	96.2
No	16	3.8
**Duration on ART (N = 407)**		
1–12 month	180	42.5
≥13month	227	53.7
**Partner’s HIV status**		
Negative	118	27.9
Positive	119	28.1
I do not know	186	44.0
**Discuss with sexual partner for HIV testing**		
Yes	112	26.5
No	311	73.5
**Partner’s reaction for HIV testing(N = 112)**		
Get angry	12	2.8
Was happy	48	11.3
Reluctant to discuss	52	12.3
**Seen HIV-positive person**		
Yes	378	89.4
No	45	10.6
**Association member of PLWHA**		
Yes	37	8.7
No	386	91.3
**Infected any family member/s with HIV**		
Yes	25	5.9
No	398	94.1
**Knowledge**		
Good knowledge	222	52.5
Poor knowledge	201	47.5
**Attitude**		
Favorable attitude	370	87.5
Poor attitude	53	12.5

^b^ = with their mother, sister, brother, friends, PLWHIV, People Living with human Immune Deficiency Virus, HIV; Human Immunodeficiency Virus, ART; Antiretroviral Therapy

### Disclosure of HIV positive status to the sexual partner

The overall prevalence of pregnant women disclosing their HIV status to their sexual partner was 73.0% (95% CI: 68.9%, 77.3%) and to anyone was 79.4% (95% CI: 75.2%, 83.5%). The women were asked why not disclose their HIV status, the most common reasons for not disclosing their HIV statuses were fear of stigma and rejection (38.7%) and fear of confidentiality (21.9%). After disclosure, one-thirds (44.7%) of women gained their sexual partners’ support. Of the women who revealed their HIV status to others, 32.7% of them disclose to their mothers [Table 4].

**Table 4:** Disclosure of HIV-positive status and reasons for not disclosing HIV status to their sexual partner among HIV-positive pregnant women in Amhara Region Referral Hospitals, Ethiopia.

Characteristics	Frequency	Percent
**Disclosed HIV status to their current sexual partner(N = 423)**		
Yes	309	73.0
No	114	27
**Reason for not disclosing (N = 114)**		
Fear of stigma and rejection	44	38.7
Fear of confidentially	25	21.9
Fear of abandonment	23	20.2
Psychological factor	17	14.9
Fear of accusation of infidelity	5	4.3
**Partner reaction after disclosure (N = 309)**		
Was supportive	138	44.7
Neutral	99	32.1
Decided to be tested	40	12.9
Anger	22	7.1
Stigma and discrimination	10	3.2
**Disclosure of HIV-status to others(N = 423)**		
Yes	336	79.4
No	87	20.6
**Disclosure of HIV- status for whom (N = 336)**		
Mother	110	32.7
Father	88	26.3
Friends	51	15.2
Other family members	41	12.2
Relatives	20	5.9
Child/children	15	4.4
Others ^c^	11	3.3

^c^ = religious leaders, neighbors

HIV; Human Immunodeficiency Virus

### Factors associated with pregnant women’s HIV status disclosure to sexual partners

On univariable logistic regression, being an urban resident, women’s having higher educational status, women’s having higher family monthly income, planned pregnancy, diagnosed HIV before pregnancy, currently on ART, disclosing their HIV status to others, knowledge, and attitude of women towards HIV/AIDS were had an association to disclose HIV status to sexual partners. However, on the multivariable logistic regression analysis urban residence, planned pregnancy, diagnosed HIV before pregnancy, disclosure of HIV status to others, and being knowledgeable on HIV transmission, and prevention were independently associated factors for disclosing their HIV status to their sexual partner.

The odds of pregnant women’s HIV disclosure status to their sexual partner were 5.04 times higher among women living in the urban setting as compared to their rural counterparts (AOR = 5.04, 95% CI, 2.14, 11.81). Likewise, the odds of pregnant women’s HIV disclosure status to their sexual partner among women whose pregnancy was planned was 2.46 times compared to women whose pregnancy was unplanned (AOR = 2.46, 95% CI:1.32, 4.57).

This study found that the odds of pregnant women’s HIV status disclosure to their sexual partner was 7.77 times higher among women who knew their HIV status before pregnancy as compared to women who were diagnosed during pregnancy (AOR = 7.77, 95% CI: 3.09, 19.52).

This study also found that pregnant women’s HIV status disclosure to their sexual partner among women who disclose their HIV status to anyone was 7.01 times higher compared to women who had not disclosed to others (AOR = 7.01, 95% CI: 3.78, 13.25).

Lastly, the odds of pregnant women’s HIV status disclosure to their sexual partner among women who had good knowledge on HIV transmission and prevention were 2.19 times higher as compared to women who had poor knowledge (AOR = 2.19, 95% CI: 1.22, 3.94) [Table 5].

**Table 5:** Bi-variable and multivariable logistic regression analysis of factors associated with HIV status disclosure to sexual partners among HIV-positive pregnant women in Amhara Region Referral Hospitals, Ethiopia.

Variables	Disclosure of HIV status to a sexual partner		COR (95%CI)	AOR (95%CI)	p-value
	Yes	No			
**Residence**					
Urban	269	70	4.22 (2.55, 6.98)	5.04 (2.14, 11.81) **	0.000
Rural	40	44	1	1	
**Educational status of the mother**					
Cannot read and write	35	20	1	1	
Can read and write	53	20	1.51 (0.71, 3.21)	2.48 (0.97, 6.57)	0.067
Primary (1–8)	59	17	1.98 (0.91, 4.28)	1.16 (0.40, 3.36)	0.779
Secondary (9–12)	83	46	1.03 (0.53, 1.98)	0.45 (0.160, 1.30)	0.144
College and above	79	11	4.10 (1.77, 9.47)	1.37 (0.41, 4.55)	0.607
**Average monthly income of the family**					
≤ 600 ETB	99	47	1	1	
601–1601 ETB	46	28	0.78 (0.43, 1.39)	0.94 (0.40, 2.18)	0.883
1602–2602 ETB	67	24	1.33 (0.74, 2.37)	0.67 (0.27, 1.63)	0.375
≥ 2603 ETB	97	15	3.07 (1.61, 5.85)	0.97 (0.35, 2.67)	0.954
**Planned pregnancy**					
Yes	259	70	3.26 (2.00, 5.28)	2.46 (1.32, 4.57) *	0.004
No	50	44	1	1	
**Time of tested positive for HIV status**					
During pregnancy	203	108	1	1	
Before pregnancy	106	6	9.39 (3.99, 22.1)	7.77 (3.09, 19.52) **	0.000
**Currently on ART**					
Yes	302	105	3.70 (1.34, 10.2)	2.98 (0.79, 11.25)	0.107
No	7	9	1	1	
**Disclosure of HIV status to others**					
Yes	274	62	6.57 (3.95, 10.9)	7.01 (3.78, 13.25) **	0.000
No	35	52	1	1	
**Knowledge**					
Good knowledge	188	34	3.65 (2.30, 5.80)	2.19 (1.22, 3.94) *	0.009
Poor knowledge	121	80	1	1	
**Attitude**					
Poor attitude	26	27	1	1	
Favorable attitude	283	87	3.38 (1.87, 6.09)	1.46 (0.67, 3.18)	0.34

AOR = Adjusted odd ratio, COR = Crude odd ratio, CI = Confidence interval, HIV = Human Immune virus, ART = Antiretroviral Therapy, ETB = Ethiopian Birr, 1 = Reference category,

* P< 0.05, and

** P ≤ 0.001

## Discussion

This study assessed HIV status disclosure to sexual partners and associated factors among pregnant women living with HIV in Amhara region referral hospitals. The prevalence of HIV status disclosure to their sexual partners among pregnant women living with HIV was 73.0%. The finding of this study is lower compared with earlier studies conducted in Nigeria-88% [32], Northwest Ethiopia-89.7% [14], Dire Dawa, Ethiopia-86.5% [27]. The possible discrepancy from those studies may be due to difference in the socio-demographic characteristics of the study subjects. Nigeria and Northwest Ethiopia studies were conducted in the urban population only [32]. This might be because of easy access to information and treatment opportunities compared to the people living in rural areas [16]. Additionally, the study participants in Northwest Ethiopia were married and knew sexual partners’ HIV positive status [14]. This might be because married women feel responsible for their husband and children to defend from HIV infection and women knew partner’s HIV positive status might not fear the negative consequences disclosure [12, 14, 27]. The other discrepancy from Dire Dawa, Ethiopia study might be different in educational level in which nearly half of the study participants completed secondary education, and over half of the participants had gotten counseling before the test [27]. Education and counseling before the HIV test were vital to bringing behavioral change and increases in awareness levels among women [33]. This might result in information variation about the importance of HIV status disclosure among the study population.

However, this result is comparable with studies conducted in Tanzania-69.6% [34], and studies elsewhere in Ethiopia including Addis Ababa-73% [13], western Ethiopia-71.2% [35], and Southwest Ethiopia-69% [36]. On the contrary, this result is higher compared with other studies conducted in Uganda-57% [16], South Africa-58.4% [37], and from two studies in Ethiopia, Addis Ababa-51.7% [12], and Mekelle-63.8% [33]. This difference might be because of variations in the different study settings, study period, study population. Most of study participants in Uganda were polygamous [16]. This might be because this group of women well-thought-out the disclosure process to be difficult. The South African study was conducted among women only living in the rural setting. Women who live in the rural areas may not have easy access to information, and all kind of services than counterparts [38]. The current study included specifically only pregnant women living with HIV while the studies conducted in Mekelle [33] included non-pregnant women. These might influence the disclosure of HIV status Additionally, the discrepancy from the Addis Ababa study might be the study period [12]. It was conducted in a fleeting period compared with the present study. Therefore, it might be, the extended study period result that there is encouraging disclosure by health care professionals, and improvement issues related to stigma and discrimination, the existence of advanced PMTCT, and antiretroviral treatment programs currently.

Regarding factors affecting HIV serostatus disclosure to a sexual partner, this study found that being an urban resident was a significant predictor of pregnant women’s HIV status disclosure to their sexual partners. In this study, those pregnant women living in urban area was 5.04 times more likely to disclose their HIV status to their sexual partner than their rural counterparts. This study is supported by previous studies from Uganda [16], and Nekemte, western Ethiopia [35]. The probable reason might be women living in the urban areas had more exposure to mass media and easily access almost all kinds of services and Information [38, 39]. Information has a significant impact in ensuring the development of behavioral change [38]. Gaining continuous information helps patients develop healthy behavior that leads to an open discussion with their sexual partners. The women also prioritize their health over social opinions, cultural influence, stigma, and discrimination and are more efficient in explaining the potential health risks to improve their healthy life expectancy. This calls upon governmental, and other non-governmental organizations to give a great emphasis on the access available services in the rural parts to achieve the national goals.

The odds of HIV status disclosure to their sexual partners among women who had planned pregnancy was 2.46 times higher as compared to those women who had an unplanned pregnancy. This study is consistent with a previous study done in Zambia [40]. A possible explanation could be the disclosure of HIV status to their sexual partner might be easy in planned pregnancy. Because, if the pregnancy is planned, they might have a discussion with their partner on the potential health risks including fear of the child might encounter HIV, medical care, and think to protect the family from the virus. Whereas unplanned pregnancy is associated with more negative reactions by partners after disclosure like worries, depression, and sadness [40]. According to evidence support, women who have planned pregnancy are more likely to have a good health-seeking intention and able to use maternal health services like ANC [41]. Hence, if women visit health facilities for healthcare use, they will have an open discussion with healthcare providers about the need for HIV status disclosure and good adherence to ART drugs [33]. Besides, women may use the maternal continuum of care and getting continuous counseling from healthcare providers that in turn, help women to outweigh the benefits and risk of not disclosing their HIV status to their sexual partners [9]. This incites provide to genuinely addressee to maternal counseling about planning pregnancy throughout the maternal continuum of care.

So, women who knew their HIV status before the last pregnancy were 7.77 times more likely to disclose to HIV status to their sexual partner than women who were diagnosed during the most recent pregnancy. This finding is similar to the study done in Tanzania [21]. Women had enough time to think and come to an agreement to receive their status. Therefore, its results are easier to disclose than those who were recently diagnosed. The other reason as evidence supports that, women who stay longer duration in HIV care services will inform about the benefits of disclosure, experience sharing with others, and got ongoing counseling. This, in turn, will encourage the women to disclose their HIV status to their sexual partners [33]. Another plausible reason might be that newly diagnosed pregnant women have been attributed to post-traumatic stress disorder following HIV diagnosis [42]. This occurs among many women testing HIV positive for the first time in pregnancy and was found to be depressed. This could be an extremely upsetting event in which being pregnant and HIV positive almost around the same time [37]. Therefore, they might not be emotionally ready to disclose, due to fear of negative consequences [43]. This indicates that the healthcare providers counsel deeply the advantage of disclosing their HIV status at the time of testing.

Disclosing their HIV status to anyone was a crucial factor in women’s HIV status disclosure to their sexual partners in the current study. Pregnant women who disclosed their HIV status to anyone were 7.01 times more likely to disclose their HIV status to their sexual partner as compared to women who had not disclose to anyone. No study supports this finding. Evidence supports that many PLWHA are getting confused and it is a big concern how, and for whom they will disclose their HIV status. Telling how they feel to someone really could be helpful and maybe a reliable source of support [44]. Besides, joint discussion with others will help them to obtain supportive ideas or information for the benefit of disclosing HIV status to their parents and their sexual partners. They might even access experiences of expression as well. This showed that health care providers should be advised of the advantage of disclosing their HIV status to someone though out the maternal continuum of care.

Lastly, the current study has proven that being knowledgeable on HIV transmission and prevention was a significant factor associated positively with HIV status disclosure to their sexual partners. Hence, pregnant women who had good knowledge of HIV transmission and prevention were 2.19 times more likely to disclose HIV serostatus to their sexual partners compared with those women who had poor knowledge. This study is consistent with a previous study done in Malaysia [45]. The likely reason might be, knowledge of HIV transmission is a precondition to prevent HIV infections [46]. Women who have good knowledge on transmission and prevention of HIV/AIDS, are more confident to explore their HIV status and prioritize their health over societal opinions and more efficient in explaining to their partners the potential health risks including fear of the child might encounter HIV and knowledgeable women might have an open discussion with their partner on medical care, and treatment, to protect the family from the virus, as well as how to improve their life expectancy. This in turn provokes providers to awareness creation strategies to improve knowledge on HIV in various aspects especially in on transmission and prevention of HIV/AIDS.

### Strength and limitation of the study

The 100% response rate supported to investigate multifactorial nature of disclosure. In general, this study came up with several independent contributing factors that have policy implications and need to give more emphasis in the findings to reduce the incidence of the disease.

Regarding the limitation, HIV disclosure status was assessed at one point in time; however, disclosure is a process and not a single event. This might underestimate the prevalence of HIV status disclosure. The data was collected through face-to-face interview. Thus, social desirability bias might have occurred, and the participants may overstate the prevalence of HIV disclosure. However, since patients were counselled that their responses will not affect their care in the hospital and that their responses are anonymous, coupled with the fact that the data collectors were not members of staff, we believe our result and conclusion is valid. Additionally, only content and face validation were done for tool, but not statistical validation.

## Conclusion

The prevalence of HIV-positive status disclosure to sexual partners was high. Being urban residents, having planned pregnancy, being HIV diagnosed before pregnancy, disclosing their serostatus to others, and women who had good knowledge on transmission and prevention of HIV/AIDS were found to be factors associated with HIV status disclosure to their sexual partners. Therefore, setting strategies in preventing unplanned pregnancy, HIV diagnosed before pregnancy, and increasing knowledge of HIV prevention will have significant role in mounting women’s disclosure status.

## Supporting information

S1 File Amharic and English version of the questionnaire. (DOCX)

S2 File SPSS dataset. (SAV)

## Abbreviations

AIDS: acquired immune deficiency Syndrome
ANC: Antenatal Care
AOR: Adjusted Odds Ratio
ART: Antiretroviral Therapy
CI: Confidence Interval
COR: Crude Odds Ratio
ETB: Ethiopian Birr
HIV: Human Immunodeficiency Virus
NGO: Non-governmental Organization
PLWHA: People Living with Acquired Immune Deficiency Virus
PMTCT: Prevention of Mother to Child Transmission
SPSS: Statistical Package for Social Science
