# Supplementary material for: Disclosure of HIV status to sexual partner and its associated factors among pregnant women living with HIV attending prenatal care in Amhara Regional state Referral Hospitals, Ethiopia
Source: PLoS One. 2023 Jan 17;18(1):e0280045. doi: 10.1371/journal.pone.0280045 (PMC9844863; doi:10.1371/journal.pone.0280045)
Supplement: S1 File — (DOCX) [file pone.0280045.s001.docx]

Annex III: English version of the questionnaires’

**Identification number……………...**

| Section I socio-demographic Information | | | | | | | | |
| --- | --- | --- | --- | --- | --- | --- | --- | --- |
| SN | Questions | Response | | | | | Skip | |
| 101 | How old are you? | ______________ Years | | | | |  | |
| 102 | Where is your permanent residence? | 1. Rural  2. Urban: | | | | |  | |
| 103 | What is your major occupation currently?  (Whatever you do to earn money)? | 1. Government employee  2. Employee of private organization/enterprise  3. NGO employee  4. merchant  5. Farmer  6. Housewife  7. Other specify ___________ | | | | |  | |
| 104 | What is your Educational level? | 1.can’t read and write  2.can read and write  3.primary(1-8)  4. secondary(9-12)  5. college and above | | | | |  | |
| 105 | What is your religion? | 1. Orthodox 2. Muslim 3. Protestant 4. Catholic 5. Other, Specify ____________ | | | | |  | |
| 106 | What is your current marital status? | 1. Single  2. married  3.separated  4. divorced  5. widowed | | | | | If 1,4, and 5 go to Q  108 | |
| 107 | What Is your sexual partner’s educational status? | 1.can’t read and write  2.can read and write  3. primary(1-8)  4. secondary(9-12)  5.college and above | | | | |  | |
| 108 | What is your sexual partner’s major occupation currently? | 1. Government employee 2. NGO employee 3. Private organization 4. merchant 5. Farmer 6. Daily laborer 7. Other specify ___________ | | | | |  | |
| 109 | What is the average family monthly income? | ________ Birr/month | | | | |  | |
| Section II social characteristics | | | | | | | | |
| 201 | Have you ever seen HIV positive person | 1. Yes 2. No | | | | | |  |
| 202 | Are you a member of any association of PLWHA? | 1. Yes 2. No | | | | | |  |
| 203 | Have you seen the mistreatment of PLWHA | 1.yes  2.no | | | | | |  |
| 204 | Is there anyone else in your family who is HIV infected? | 1.yes  2.no | | | | | |  |
| Section III obstetrics characteristics | | | | | | | | |
| 301 | Number of pregnancy |  | | | | | |  |
| 302 | How many alive children do have? | ----------------- | | | | | |  |
| 303 | Is the pregnancy planned? | 1. Yes 2. NO | | | | | |  |
| 304 | Is the pregnancy wanted? | 1. Yes 2. NO | | | | | |  |
| 305 | Your current ANC visit | 1.first  2.second  3.third  4.fourth or more | | | | | |  |
| Section iv Clinical characteristics | | | | | | | |  |
| 401 | When did you diagnose your HIV? | 1. During pregnancy 2. Before pregnancy | | | | | |  |
| 402 | How much time has passed since testing for HIV? | 1. less than 6 months  2. 6 months and more | | | | | |  |
| 403 | Where were you tested? | 1. government Health facility  2. private clinic  3. Free standing VCT centers  4. Others(specify)__________________ | | | | | |  |
| 404 | Did you get counseled before a test? | 1. Yes 2. No | | | | | |  |
| 405 | Did you get counseled after the test? | 1. Yes 2. No | | | | | |  |
| 406 | Did you get ongoing counseling when you visit the health facility | 1. Yes 2. No | | | | | |  |
| 407 | With whom did you test your blood | 1. Alone 2. With my Sexual partner 3. Other specify__________________ | | | | | |  |
| 408 | Are you currently on ART? | 1. Yes 2. no | | | | | |  |
| 409 | Duration of ART | --------month/years | | | | | |  |
| 410 | What do you know about your partner’s HIV status? | 1. HIV positive 2. HIV negative 3. I do not know | | | | | |  |
| 411 | Have you ever talked about HIV testing with your Sexual partner before getting tested? | 1. Yes  2. No | | | | | |  |
| 412 | What was his reaction to the discussion on HIV testing? | 1.Was happy  2. Was reluctant to discuss  3. Get angry  4. Other | | | | | |  |
| Section V. Disclosure of HIV positive status and reasons | | | | | | | | |
| 501 | Have you disclosed your HIV results to your current sexual partner? | 1. Yes 2. No | | | | | | Skip to Q.502 |
| 502 | Reasons? why you didn’t tell your partner? | 1. Fear of stigma and rejection 2. Psychological factor 3. Fear of abandonment 4. Fear of confidentially 5. Fear of accusation of infidelity 6. Other(Specify)____________________ | | | | | |  |
| 503 | What was your partner's reaction when he knew that you are HIV positive? | 1. Was supportive  2. Neutral  3. Decided to be tested  4. anger  5. Stigma and discrimination  6.violence  7. Other (specify)__________________ | | | | | |  |
| 504 | Have you disclosed your HIV status to anyone (others)? | 1.yes  2.no | | | | | |  |
| 505 | If yes, whom have you told you are HIV positive?  (Multiple responses possible) | 1. Mother 2. Father 3. Child 4. Other Family members 5. Relatives 6. Friends 7. Other---------- | | | | | |  |
| Section vi Knowledge on HIV transmission and prevention to HIV/AIDS | | | Yes | | no | Don’t know | |  |
| 601 | Consistent condom use can prevent the transmission of HIV | | 1 | | 2 | 3 | |  |
| 602 | Limiting with one uninfected sexual partner prevent HIV infection | | 1 | | 2 | 3 | |  |
| 603 | HIV can spread by mosquitoes | | 1 | | 2 | 3 | |  |
| 604 | A person can get HIV by sharing a meal with an infected person, | | 1 | | 2 | 3 | |  |
| 605 | A healthy-looking person can have HIV/AIDS | | 1 | | 2 | 3 | |  |
| Vii | Attitudes of HIV positive pregnant women to HIV/AIDS | | | | | | | |
|  |  | | Strongly agree | Agree | Neutral | disagree | | Strongly disagree |
| 701 | Being HIV positive is not the end of life | | 5 | 4 | 3 | 2 | | 1 |
| 702 | If an HIV /AIDS positive person wants to have he or she should always talk about safe sex with his/her partner | | 5 | 4 | 3 | 2 | | 1 |
| 703 | An HIV-positive person who decides to have sex should always use a condom. | | 5 | 4 | 3 | 2 | | 1 |
| 704 | It is shameful to have HIV/AIDS | | 1 | 2 | 3 | 4 | | 5 |
| 705 | Asexual/spouse of HIV positive person should get tested | | 5 | 4 | 3 | 2 | | 1 |

**The end. Thank you very much**!

Annex VI: አማርኛ ቃለ መጠይቅ

የመለያ ቁጥር……………..

| **ክፍል ፣ የተሳታፊዋ አድራሻና የማህበራዊ ኑሮ ሁኔታ** | | | | | | | | | | | |
| --- | --- | --- | --- | --- | --- | --- | --- | --- | --- | --- | --- |
| **ተ.ቁ** | **ጥያቄ** | **ምላሽ** | | | | | | **የሚታለፍ** | | | |
| 101 | እድሜሽ ሰንት ነው? | ----------------------------ዓመት | | | | | |  | | | |
| 102 | ቋሚ የመኖሪያ ቦታሽ የት ነው? | 1. ገጠር 2. ከተማ | | | | | |  | | | |
| 103 | በአሁኑ ጊዜ ዋናው ስራሽ ምንድን ነው?  ( ማነኛውም ስራ ገቢ ምታገኝበት) | 1. የመንግስት ሰራተኛ  2. የግ ድርጅት ተቀጣሪ  3 መንግስታዊ ያልሆን ድርጅት ተቀጣሪ  4 ነጋዴ  5. ገበሬ  6. የቤት እመቤት  7. ሌላ (ይጠቀስ) | | | | | |  | | | |
| 104 | የትምህርት ደረጃ ስንት ነው | 1. ማንበብና መጻፍ የማይችል  2. ማንበብና መጻፍ የሚችል  3. የመጀመርያ ደርጃ (1-8)  4. የሁለተኛ ደረጃ(9-12)  5.ኮሌጅ እና ከዛ በላይ | | | | | |  | | | |
| 105 | ሐይማኖትሽ ምንድን ነው? | 1. ኦርቶዶክስ  2. ሙስሊም  3. ፕሮቴስታንት  4. ካቶሊክ  5. ሌላ (ይጠቀስ) ------------- | | | | | |  | | | |
| 106 | በኣሁኑ ጊዜ የትዳር ሁኔታ እነዴት ነው ? | 1. ኣላገባሁም (የወነድ ጓደኛ አለኝ)  2. አግብቻለሁ አብርን ነን  3. ተጋብተን በተለያየ ቦታ ነው የምንኖረው  4. ተለያይተናል(ተፋተናል)  5. ሙቶብኛል | | | | | | መልስዎ 1፣4፣ዕና 5 ከሆነ  ወደ ጥ.ቁ.109 ሂድ | | | |
| 107 | የአሁኑ ባለቤትሽ(ጾታዊ ጓደኛሽ) የትምህርት ደረጃዉ ስንት ነው? | 1. ማንበብና መጻፍ የማይችል  2. ማንበብና መጻፍ የሚችል  3. የመጀመርያ ደርጃ (1-8)  4. የሁለተኛ ደረጃ(9-12)  5.ኮሌጅ እና ከዛ በላይ | | | | | |  | | | |
| 108 | የአሁኑ ባለቤትሽ(ጾታዊ ጓደኛሽ) ዋና ስራ ምንድን ነው ? | 1. የመንግስት ሰራተኛ  2. መንግስታዊ ያልሆን ድረጅት ተቀጣሪ  3 የግል ድረግት ተቀጣሪ  4. ነጋዴ  5. ገበሬ  6. የቀን ሰራተኛ  7. ሌላ (ይጠቀስ) | | | | | |  | | | |
| 109 | የቤተሰቡ አማከኝ የወር ገቢ ስንት ነዉ? | ________________________ | | | | | |  | | | |
| ክፍል. 2 የማህበራዊና ተያያዥ ጥያቄዎች | | | | | | | | | | | |
| 201 | ኤች አይ ቪ ፖዘቲቭ ሰዉ አይተሸል? | 1. አዎ 2. የለም | | | | | | |  | | |
| 202 | ከቫይረሱ ጋራ ከሚኖሩ ወገኖች አባል ነሽ ? | 1. አዎ  2. አይደለሁም | | | | | | |  | | |
| 203 | በቤተሰብ ዉሰጥ ሌላ ከቫይረሱ ጋር የሚኖር ሰው አለ? | 1. አዎ  2 . የለም | | | |  | | | | |  |
| 204 | ኤች አይ ቪ ያለባቸው ሰዎች ተፅዕኖ ሲደረግባቸው አይተሸል? | 1.አዎ  2. የለም | | | |  | | | | |  |
| ክፍል.3 የእርግዝና እና ተያያዥ ሁኔታ | | | | | | | | | | | |
| 301 | ያሁኑ እርግዝናሸ ስንተኛሽ ነዉ? | --------- | | | | | | |  | | |
| 302 | ባሁኑ ሰኣት ስንት ልጅ/ልጆች ኣሉሽ? | ------------- | | | | | | |  | | |
| 303 | እርግዝናዉ አቅደሽ ያረገዝሽዉ? | 1. ኣዎ 2. አይ | | | | | | |  | | |
| 304 | እርግዝናዉ ፈልገሽ ነዉ ያረገዝሽዉ? | 1. አወ  2. ሳልፈልገዉ ነዉ | | | | | | |  | | |
| 305 | ኣሁን ስነተኛ ክትትልሽ ነዉ? | 1.የመጀመሪያ  2.ሁለተኛ  3.ሶስትኛ  4.አራት እና በላይ | | | | | | |  | | |
| ክፍል 4 ኤች አይ ቪ የደም ምርመራ እና ተያያዥ ሁኔታ | | | | | | | | | | | |
| 401 | ስትመረመሪ ነፍሰጡር ነበረሽ ? | 1. ኣዎ  2. አልነበርሁም | | | | | | |  | | |
| 402 | ምንያክል ጊዜ ሆነሽ የኤቺ አይ ቪ የደም ምርመራ ከተመረመርሽ? | 1.ከ6 ወር በታች  2. 6 ወርና በላይ | | | | | | |  | | |
| 403 | የት ነዉ የተመረመርሺዉ? | 1. ከመንግስት ጤና ተቃም  2. ከግል ከሊኒክ  3. በነጻ ምርመራ አገልግሎት ከሚሰጡ ቦታዎቸ  4.ሌላ ካለ ይጥቀሱ-------------------------- | | | | | | |  | | |
| 404 | ምርመራ ከማድረግዎ በፊት ምክር አግኝተዉ ነበር ? | 1.አዎ  2. አላገኘሁም | | | | | | |  | | |
| 405 | ምርመራ ካደረጉ በኋላ ምክር አግኝተዉ ነበር ? | 1.አዎ  2. አላገኘሁም | | | | | | |  | | |
| 406 | ወደ ጤና ጣቢያ ሲመጡ ሁሌ የምክር አገልግሎት ያገኛሉ? | 1. አዎ  2. አላገኝም | | | | | | |  | | |
| 407 | ምርመራ ሲያደርጉ ከማን ጋር ነበሩ? | 1.ብቻየን  2. ከጓደገኛ ጋር  3.ሌላ ካለ ይጥቀሱ------------- | | | | | | |  | | |
| 408 | በአሁኑ ጊዜ የጸረ ኤች አይ ቪ መድሃኒት ትወስጃለሽ ? | 1. አዎ  2 . አለወስድም | | | | | | |  | | |
| 409 | ምነ ያክል ጊዜ ሆንዉ ከጀመርሽ? | ----------ወር/አመት | | | | | | |  | | |
| 410 | ባንች አመለካከት የጾታዊ ጓደኛሽ) የደም ምርመራ ውጤት ምን ይመስላል ? | 1. ኤች አይ ቪ ፖዘቲቭ  2. ኤች አይ ቪ ነጌቲቭ  3. አላውቅም | | | | | | |  | | |
| 411 | ከመመርመርሽ በፊት ከባለቤትሽ(ጾታዊ ጓደኛሽ) ጋር ስለምርመራ ተነጋረሽ ታቂያለሽ? | 1. አዎ  2. አናውቅም | | | | | | |  | | |
| 412 | ከተነጋገራችሁ የሱ ምላሽ ምን ነበር? | 1. ተደሰተ  2. ችላ አለ  3. ተናደደ  4. ሌላ ይጠቀስ--------- | | | | | | |  | | |
| ክፍል.5 የኤች አይቪ የደም ምርመራ ውጤትን ለሌሎች መናገር ወጤቱና ምክንያቶቹ፡፡ | | | | | | | | | | | |
| 501 | ከቫይረሱ ጋር እንደምትኖሪ አሁኑ ላለዉ ጾታዊ ጓደኛሽ ነግረሽዋል? | 1 አዎ  2. አልተናገርኩም | | | | | | | ወደጥ.502 | | |
| 502 | የምርመራ ውጤትሽን ካልተናገርሽ ለምን አልተናገርሽም ? | 1. መገለልና ጥላቻ ይደረስብኛል ብየ ስለምፈራ  2 .የስነልቦና ችግር ስላለብኝ  3. ትቶኝ ይሄዳል ብየ ስለምፈራ  4. ሚስጥር ይወጣል ብየ ስለምፈራ  5. እምነቴን አጉድያለሁ ብየ ስለማስብ  6. ሌላ (ይጠቀስ) ------------ | | | | | | |  | | |
| 503 | ባለቤትሽ ውጤትሽን በሰማ ወቅት ምላሹ ምን ነበር ? | 1. አጽናናኝ  2. ምንም አላለም  3.ለመመርመር ወሰነ  4. ተናደደብኝ  5. ጥላቻ እና መድሎ አደረገብኝ  6. ጥቃት አደረሰብኝ  7. ሌላ (ይጠቀስ) ___________________ | | | | | | |  | | |
| 504 | ውጤትሽን ለሌላ ሰው ተናግረሻል? | 1. አዎ 2. አይ | | | | | | |  | | |
| 505 | ከነገርሽ ለማን ነው የተናገርሽ?  (ከአንድ በላይ መስጠት ይቻላል): | 1. ለእናቴ  2. ለአባቴ  3. ለልጄ  4. ሌላ የቤተሰብ አባል  5. ለዘመዶቼ  6. ለጓደኞቼ  7 ሌላ(ይጠቀስ)----- | | | | | | |  | | |
| \| ክፍል 6. የኤች አይ ቪ ኤዲስ መተላለፊያና መከላከያ መንገዶች የማወቅ ችሎታ \|  \| \| --- \| --- \| | | | | | | | | | | | |
| 601 | ኮንዶምን ባግባቡ መጠቀም ኤች አይ ቪ እንዳይተላለፍ ይረዳል | | 1.አዎ | 2.አይረዳም | | | 3. አላዉቅም | | |  | |
| 602 | በሽታዉ ከሌለበት ሰዉ ጋር አንድ ለአንድ መወሰን በሽታዉ እነዳደይዘን ይረዳል | | 1.አዎ | 2.አይረዳም | | | 3. አላዉቅም | | |  | |
| 603 | የኤቸ አይ ቪ በወባ ትንኝ ይተላለፋል? | | 1. ኣዎ | 2. አይተላለፍም | | | 3. አላዉቅም | | |  | |
| 604 | ጤነኛ መስሎ ሚታይ ሰዉ ኤች አይ ቪ ሊኖረበት ይችላል | | 1.አዎ | 2.አይኖረዉም | | | 3. አላዉቅም | | |  | |
| 605 | ኤች አይ ቪ በሽታዉ ካለበት ሰዉ ጋር አብሮ በመብላት ይተላለፋል | | 1.አዎ | 2. አይተላለፍም | | | 3. አላዉቅም | | |  | |
| \| ክፍል \| 7. ስለ የኤች አይ ቪ ኤዲስ ያላቸዉን አመለካከት ለማወቅ \| \| --- \| --- \| | | | | | | | | | | | |
|  |  | | በጣም እስማማለሁ | እስማማለሁ | አላዉቅም | | አልስማማም | | | በጣም አልስማማም | |
| 701 | ኤች አይ ቪ ፖዘቲቭ መሆን መሞት አደለም | | 5 | 4 | 3 | | 2 | | | 1 | |
| 702 | ኤች ፖዘቲቭ የሆነ ሰዉ ሁሌም ከጓደኛዉ ጋር ስለጾታዊ ግነኙነት መነጋገር አለባቸዉ | | 5 | 4 | 3 | | 2 | | | 1 | |
| 703 | ኤች አይ ቪ ፖዘቲቭ የሆነ ሰዉ ሑሌም ኮነዶም መጠቀም አለበት | | 5 | 4 | 3 | | 2 | | | 1 | |
| 704 | በኤች አይ ቪ ኤዲስ መያዝ አሳፋሪ ነዉ | | 1 | 2 | 3 | | 4 | | | 5 | |
| 705 | ጓደኛዉ ኤች ኣይ ቪ ፖዘቲቭ የሆነ ሰዉ የደም ምርመራ ማድረግ አለበት | | 5 | 4 | 3 | | 2 | | | 1 | |
| ቃለ መጠይቁ አበቃ! ስለትብብርዎ በጣም አመሰግናለሁ | | | | | | | | | | | |
